# Genome re-assignment of *Arachis trinitensis* (Sect. *Arachis*, Leguminosae) and its implications for the genetic origin of cultivated peanut

**DOI:** 10.1590/S1415-47572010005000079

**Published:** 2010-12-01

**Authors:** Germán Robledo, Graciela I. Lavia, Guillermo Seijo

**Affiliations:** 1 Instituto de Botánica del Nordeste, CorrientesArgentina; 2 Facultad de Ciencias Exactas y Naturales y Agrimensura, Universidad Nacional del Nordeste, CorrientesArgentina; 3 Facultad de Ciencias Agrarias, Universidad Nacional del Nordeste, CorrientesArgentina

**Keywords:** CMA/DAPI bands, genome donors, peanut genetic origin, rDNA loci

## Abstract

The karyotype structure of *Arachis trinitensis* was studied by conventional Feulgen staining, CMA/DAPI banding and rDNA loci detection by fluorescence *in situ* hybridization (FISH) in order to establish its genome status and test the hypothesis that this species is a genome donor of cultivated peanut. Conventional staining revealed that the karyotype lacked the small “A chromosomes” characteristic of the A genome. In agreement with this, chromosomal banding showed that none of the chromosomes had the large centromeric bands expected for A chromosomes. FISH revealed one pair each of 5S and 45S rDNA loci, located in different medium-sized metacentric chromosomes. Collectively, these results suggest that *A. trinitensis* should be removed from the A genome and be considered as a B or non-A genome species. The pattern of heterochromatic bands and rDNA loci of *A. trinitensis* differ markedly from any of the complements of *A. hypogaea*, suggesting that the former species is unlikely to be one of the wild diploid progenitors of the latter.

## Introduction

The genus *Arachis* is native to South America and comprises 80 species arranged in nine sections (Krapovickas and Gregory, 1994; Valls and Simpson, 2005). Section *Arachis* is the most numerous, with 30 wild diploid species (2n = 2x = 20, 2n = 2x = 18) and two allopolyploid entities (2n = 4x = 40), *i.e.*, the cultigen *A. hypogaea* (peanut) and its presumed wild progenitor *A. monticola* (Fernández and Krapovickas, 1994; Lavia, 1996, 2000; Peñaloza and Valls, 2005; Lavia *et al.*, 2008). The geographic range of these species extends from the foothills of the Andes mountains in Bolivia and northern Argentina to the Atlantic coast in Brazil, and from the headwaters of the Mamoré and Guaporé rivers in northern Bolivia and Tocantins river in central Brazil to the northern coast of the La Plata river in Uruguay.

Based on cross-compatibility assays and chromosomal features, three genomes (A, B and D) have been designated in section *Arachis* (Smartt *et al.*, 1978; Gregory and Gregory, 1979; Singh and Moss, 1982, 1984; Singh, 1986; Stalker, 1991; Fernández and Krapovickas, 1994; Singh and Smartt, 1998; Lavia *et al.*, 2008). With the exception of *A. glandulifera* (D genome), all of the species have symmetric karyotypes (Stalker, 1991; Fernández and Krapovickcas, 1994; Lavia, 1998; Peñaloza and Valls, 2005; Lavia *et al.*, 2009) and have been assigned to the A or B genomes. The A genome group is the most numerous and consists of 16 species. The karyotypes of these taxa are characterized by the presence of a small chromosomal pair, the “A chromosomes”. These chromosomes are half the size of the longest chromosomes of the karyotype and have a diffuse coloration (except for the centromeric bands) in prophase and pro-metaphase. In general, the A genome species are karyotypically homogeneous, with similar patterns of heterochromatin distribution (Seijo *et al.*, 2004; Robledo *et al.*, 2009). The remaining species with symmetric karyotypes have traditionally been assigned to the B genome group (or non-A genome) because they lack the “A chromosomes”. These species are more diverse in their karyotype formulas (Fernández and Krapovickas, 1994; Lavia *et al.*, 2009) and heterochromatin patterns (Seijo *et al.*, 2004).

*Arachis trinitensis* (2n = 2x = 20) is an annual species that occurs in a very restricted island of cerrado-like vegetation in Trinidad, central Bolivia. This species is known from only a few individuals from each of three collection sites located in an area of less than 10 km^2^. This distribution overlaps with two other species of the section, *A. benensis* and *A. williamsii*, both of which have the B genome, but individuals of these three species have never been found in sympatry (Krapovickas and Gregory, 1994). Biogeographically, these three species are of particular interest in understanding the dispersion of the section since they occur on the north-western edge of the genus' natural range and are separated from other *Arachis* species by 200 km of seasonally flooded rainforest. For species with autogamy and geocarpic fruits like those of *Arachis*, this 200 km-wide tract of inappropriate habitat constitutes an unsurpassable barrier for gene flow and seed dispersion.

Within this group, *A. trinitensis* deserves special attention since previous chromosome reports assigned this species to the A genome group and suggested that it was a putative genome donor of the A genome of peanut (Lavia, 1996). The relative proximity of Trinidad (where some putative peanut ancestors grow) to Rurrenabaque in Beni, Bolivia (a center of high morphological diversity of *A. hypogaea*) (David Williams, personal communication) led to the proposal of an alternative center of origin for peanut. These considerations resulted in more extensive analyses of *A. trinitensis* in order to clarify its role in the genetic origin of *A. hypogaea*. A recently published analysis of *Arachis* species using amplified fragment length polymorphism (AFLP) positioned *A. trinitensis* close to *A. benensis* and other B genome group species (Milla *et al.*, 2005). However, that cluster also included *A. diogoi*, a species that belongs to the A genome. Although not conclusive, these data questioned the genome identity of *A. trinitensis* and its involvement in the genetic origin of peanut.

In view of this genome uncertainty, and since heterochromatin analysis and mapping of DNA sequences by fluorescence *in situ* hybridization (FISH) have been useful in the genomic characterization of some *Arachis* species and for studying the genetic origin of peanut (Raina and Mukai, 1999; Seijo *et al.*, 2004; Robledo and Seijo, 2008; Robledo *et al.*, 2009), we used Feulgen staining, CMA-DAPI banding and rDNA detection by FISH to reassess the genomic identity of *A. trinitensis* and to establish whether this species should still be considered a potential genome donor of *A. hypogaea*.

## Materials and Methods

###  Plant material

Three individuals from the type locality of *A. trinitensis* (Bolivia, Dept. Beni, Trinidad city, Williams 1117. CTES) were analyzed. This is the only accession that is maintained in germplasm banks, including the peanut germplasm collection of the Instituto de Botánica del Nordeste (Corrientes, Argentina).

###  Feulgen staining

Roots were obtained from seeds germinated in pots under laboratory conditions. Healthy root apices (5-10 mm long) were pre-treated with 2 mM 8-hydroxyquinoline for 3 h at room temperature (Fernández and Krapovickas, 1994) and then fixed in absolute ethanol:glacial acetic acid (3:1, v/v) for 12 h at 4 °C prior to storage at -20 °C. For conventional chromosome staining, fixed root apices were washed in distilled water for 5 min, hydrolyzed in 1 N HCl for 8 min at 60 °C, stained with Schiff's reagent (Feulgen's technique) and then squashed in a drop of 2% acetic orcein. Permanent mounts were then performed using Euparal mounting medium.

###  Fluorochrome banding

Double staining with the fluorochromes CMA-DAPI was done according to Schweizer and Ambros (1994) with modifications. Fixed root apices were digested in 1% (w/v) cellulose (from *Trichoderma viridae*; Onozuka R-10, Serva, Heidelberg, Germany) plus 10% (v/v) pectinase dissolved in 40% glycerol (from *Aspergillus niger*, Sigma, St. Louis, MO, USA), in 0.01 M citrate buffer, pH 4.8, at 37 °C for 2 h. Subsequently, the meristematic cells were removed from the root tip and squashed in 45% acetic acid. After removal of the coverslip with carbon dioxide, the slides were air-dried, aged for 1-2 days at room temperature and then kept at -20 °C until used. Chromosomal preparations were stained first with CMA (chromomycin A3, 0.5 mg/mL) for 60 min and then with DAPI (4-6-diamidino-2-phenylindole, 2 μg/mL) for 30 min, and finally mounted in McIlvaine's buffer (pH 7.0): glycerol (1:1, v/v) containing 5.5 mM MgCl_2_.

###  rDNA detection

For *in situ* hybridization, root apices were enzymatically digested as described above. The 5S rDNA and 45S rDNA loci were localized using the pA5S, and pA18S and pA26S probes, respectively, isolated from genomic DNA of *A. hypogaea* (Robledo and Seijo, 2008) and labeled by nick translation with digoxigenin-11-dUTP (Boehringer Mannheim, Mannheim, Germany) or biotin-11-dUTP (Sigma-Aldrich). The pretreatment of slides, chromosome and probe denaturation, conditions for the *in situ* hybridization (hybridization mixes contained DNA probes at a concentration of 2.5-3.5 ng/μL, with a stringency to allow sequences with 80%-85% identity to remain hybridized), post-hybridization washing, blocking and indirect detection with fluorochrome-conjugated antibodies were done according to Moscone *et al.* (1996). The first set of antibodies consisted of mouse anti-biotin (Dakopatts, Dako, Carpinteria, CA, USA) and sheep anti-digoxigenin conjugated to fluorescein isothiocyanate (FITC) (Boehringer Mannheim). The second set of antibodies consisted of rabbit anti-mouse conjugated to tetramethyl-rodamine isothiocyanate (TRITC) (Dakopatts) and FITC-conjugated rabbit antisheep (Dakopatts). The preparations were counterstained and mounted in Vectashield medium (Vector Laboratories, Burlingame, CA, USA) containing DAPI (2 mg/mL). Counterstaining with DAPI revealed a C-banding-like pattern, with major heterochromatic bands fluorescing more intensely (cf. Seijo *et al.*, 2004). Monochromatic pictures were taken for each fluorochrome, pseudocolored, and merged into one image using Leica Q-Win software.

###  Karyotype analysis

Five metaphase plates per individual were used for chromosomal measurements with the free version of MicroMeasure v. 3.3 software. The total chromosomal length of the haploid complement, mean length of each chromosome pair, centromeric index, relative length of each chromosome pair in relation to the largest chromosome pair, percentage of heterochromatin in each chromosome relative to their length, intrachromosomal asymmetry index (A1), and interchromosomal asymmetry index (A2) (Romero Zarco, 1986) were used to characterize the karyotype. The chromosomes were classified morphologically according to Levan *et al.* (1964). Data from homologous chromosomes were combined to obtain mean values, first between chromosomes in the same metaphase and then between the homologous chromosomes of all metaphases for the three individuals. The karyotype was constructed based on measurements obtained from metaphases stained by Feulgen's technique and the represented as a haploid complement in an idiogram. The chromosomes were arranged primarily by morphology and then by decreasing size. Satellite chromosomes (SAT chromosomes) were classified according to Fernández and Krapovickas (1994).

## Results

**Figure 1:**
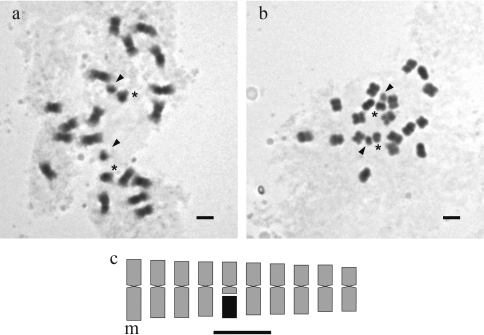
Feulgen staining and idiogram of somatic chromosomes of *Arachis trinitensis*. (A) Prometaphase showing homogeneous coloration and similar condensation pattern in all chromosomes. (B) Metaphase in which “A” chromosomes are not observed. The satellite in chromosome 10 is represented as a black block. Arrowheads in A and B indicate the satellites, and asterisks indicate the proximal segments and short arms of SAT chromosomes. (C) Idiogram with chromosomes arranged in order of decreasing size. Scale bar = 2 μm.

The karyotype of *A. trinitensis* consisted exclusively of metacentric chromosomes. Chromosomal length ranged from 1.53 to 2.13 μm and the total length of the haploid complement was 18.32 ± 1.35 μm (mean ± SD, n = 5). Chromosomal size decreased gradually and none of the pairs corresponded to the small “A chromosomes”. Intrachromosomal (A1 = 0.19) and interchromosomal (A2 = 0.10) asymmetry indices were low. All chromosomes showed homogeneous coloration and a similar condensation pattern during prophase and metaphase (Figure 1A,B). Only one pair of chromosomes with secondary constrictions was found in all of the metaphases analyzed. These constrictions were located proximally in the long arms of pair 5 (Figure 1A,B). Since the satellite was approximately the same size as the short arm, and the latter was larger than the proximal segment of the long arm (Figure 1C), SAT chromosomes were identified as type 6, according to the classification of Fernández and Krapovickas (1994).

**Figure 2:**
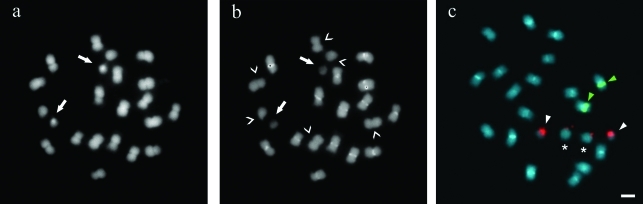
Somatic chromosomes with fluorochrome banding and rDNA detection. (A) Metaphase showing the distribution of the CMA^+^ bands in the periNOR regions (arrows). (B) Metaphase with DAPI^+^ pericentromeric bands and DAPI^-^ bands in the periNOR regions. Open arrowheads indicate chromosomes without DAPI^+^ pericentromeric bands and arrows indicate DAPI^-^ bands in the periNOR regions. (C) Somatic metaphase after double fluorescent *in situ* hybridization (FISH) showing yellow-green fluorescein isothiocyanate (FITC) signals from the 5S rDNA probe and red tetramethyl-rhodamine isothiocyanate (TRITC) signals from the 45S rDNA probe. DAPI counterstaining (light blue) subsequent to FISH was used to highlight the heterochromatin bands and to stain euchromatin. Green arrowheads indicate signals from the 5S rDNA probe, white arrowheads indicate the satellites, and asterisks indicate the proximal segments and short arms of SAT chromosomes. Scale bar = 2 μm.

Staining with fluorochromes revealed two small CMA^+^/DAPI^-^ bands located in the periNOR regions (Figure 2A) and DAPI^+^ heterochromatic bands in seven chromosome pairs (Figure 2B). The latter bands were located pericentromerically and accounted for 6.43-10.27% of the chromosomal length. The total amount of heterochromatin accounted for 5.89% of the karyotype length.

*In situ* hybridization showed one pair of 5S rDNA loci located paracentromerically on the short arms of medium-sized metacentric chromosomes, and one pair of 45S rDNA loci located proximally on the long arms of medium-sized metacentric chromosomes (Figure 2C).

## Discussion

In this study, we first evaluated the genomic identity of *A. trinitensis* by comparing its karyotype with those of species with A and B genomes, and then examined the hypothesis that this species is a putative progenitor of *A. hypogaea* by comparing the chromosome markers detected so far in each of the chromosomal complements of the cultigen with those observed in *A. trinitensis*.

###  Basis for the genome re-assignment of *A. trinitensis*

Like most of the published karyotypes for species of the section *Arachis* (Fernández and Krapovickas, 1994; Lavia, 1998, 2000; Lavia *et al.*, 2009), the karyotype of *A. trinitensis* consists of ten pairs of small to medium size chromosomes, and lacks the principal marker chromosomes that define the A genome, *i.e.*, the small “A chromosome” pair. The smallest pair of chromosomes in *A. trinitensis* had a length that corresponded to 72% of the largest chromosome pair, a value much greater than the 50% reported for “A chromosomes” (Fernández and Krapovickas, 1994). Moreover, none of the chromosomes showed allocycly, which is one of the main features used to detect “A chromosomes” in somatic prophases and pro-metaphases (Fernández and Krapovickas, 1994). Additionally, the A2 asymmetry indices for *Arachis* species in the B genome group range from 0.08 to 0.13, while those for the A genome group range from 0.13 to 0.20 (Lavia *et al.*, 2009; Robledo *et al.*, 2009). Based on these values, the A2 asymmetry index of 0.10 for *A. trinitensis* agreed best with the B genome group. Thus, despite the initial suggestion that *A. trinitensis* belongs to the A genome (Lavia, 1996), Feulgen staining of metaphases did not support this conclusion but rather indicated that this species should be re-assigned to the B or non-A genome.

Fluorochrome staining, which reveals the distribution of heterochromatin and its base composition, was very informative for assigning *A. trinitensis* to a genome group in the section *Arachis*. In all of the taxa analyzed so far, the A chromosomes have a DAPI^+^ pericentromeric band that accounts for ~45% of the chromosome length (Seijo *et al.*, 2004; Robledo *et al.*, 2009). However, in *A. trinitensis*, none of the chromosomes had heterochromatic bands that accounted for more than 10.27% of the chromosome length. This finding for chromosomal banding was consistent with results obtained by conventional staining procedures which show that the *A. trinitensis* karyotype does not contain A chromosomes.

Although the location of CMA^+^/DAPI^-^ bands coincided with the secondary constriction (as is usual in *Arachis* species), DAPI^+^ banding revealed a very specific pattern of AT-rich heterochromatin distribution in the *A. trinitensis* karyotype. To date, two general patterns of karyotypic organization have been identified among species of the B genome group: those with large pericentromeric heterochromatic bands in all chromosomes, except in one pair, and those with no detectable heterochromatic bands in the entire karyotype (Seijo *et al.*, 2004). The presence of small pericentromeric DAPI^+^ bands in seven chromosomal pairs in *A. trinitensis* is very peculiar and does not fit any of the patterns recorded to date for the B genome. The pattern described here therefore appears to be exclusive to this species and may be considered as a third karyotypic arrangement within the B or non-A genome group.

###  Evidence that *A. trinitensis* is unlikely to be a putative peanut progenitor

The fact that *A. hypogaea* has two subspecies and six botanical varieties has led to the hypothesis of different centers of origin, or at least different centers of domestication, with particular introgression events at each site. One of the traditional centers of origin is located in southeastern Bolivia and northwestern Argentina, where some populations of the wild tetraploid ancestor, *A. monticola*, still occur (Krapovickas and Gregory, 1994; Seijo *et al.*, 2004, 2007). Other proposals for peanut origin include different locations in Peru and Bolivia and consider a spontaneous origin of allopolyploids both in the wild and in the orchards of ancient agricultures (Simpson and Faries, 2001). One of these alternative sites of origin is the region of Trinidad in north-central Bolivia, where three annual species are found. This was precisely the center where *A. trinitensis*, together with *A. williamsii,* was considered a putative diploid ancestor of peanut (Lavia, 1996).

Chromosomal analysis has shown that the amphidiploid *A. hypogaea* has two easily distinguishable genomes: the A genome, in which all of the chromosomes have pericentromeric heterochromatic bands, and the B genome, which is completely devoid of these bands (Seijo *et al.*, 2004). In this context, the A genome donor must have provided all of the chromosomes with heterochromatic bands, while the B genome donor must have provided a complement without heterochromatic bands. Consequently, the presence of heterochromatic bands in seven pairs of chromosomes in *A. trinitensis* is strong evidence that this species is unlikely to have been involved in the genetic origin of peanut.

FISH analysis has shown that the A genome of *A. hypogaea* has one 5S and two 45S rDNA loci, whereas the B genome has one 5S and three 45S rDNA loci per haploid complement (Seijo *et al.*, 2004). Although a loss or gain of rDNA loci in polyploids relative to the parental plants has been observed in angiosperms (Pontes *et al.*, 2004), the *Arachis* genomes show strong quiescence after polyiploidyzation (Seijo *et al.*, 2004, 2007). Thus, the pattern found in *A. trinitensis*, with only one locus for each rDNA gene family, differs from what would be expected in the peanut genome donors.

In conclusion, the results of Feulgen staining, chromosome banding and rDNA detection indicate that *A. trinitensis* should be removed from the A genome group, and that the hypothesis that this species is a putative contributor to the peanut genome must be rejected.
